# Lactose-free milk for infants with acute gastroenteritis in a developing country: study protocol for a randomized controlled trial

**DOI:** 10.1186/s13063-015-0565-9

**Published:** 2015-02-08

**Authors:** Mona Nabulsi, Nadine Yazbeck, Fatme Charafeddine

**Affiliations:** Department of Pediatrics and Adolescent Medicine, Faculty of Medicine, American University of Beirut, Beirut, Lebanon; Department of Pediatrics and Adolescent Medicine, American University of Beirut Medical Center, P.O. Box: 113-6044/C8, Beirut, Lebanon

**Keywords:** Acute gastroenteritis, Lactose-free milk, Developing world

## Abstract

**Background:**

Acute gastroenteritis is a major cause of pediatric morbidity and mortality, accounting for 15% of all childhood deaths worldwide. In developing countries, diarrheal diseases continue to be a major public health burden. Evidence from developed countries suggests that intake of lactose-free milk during diarrheal episodes may reduce the duration of the illness in pediatric inpatients. It is unknown whether lactose-free milk reduces the severity or duration of acute gastroenteritis in infants treated in outpatient settings in developing countries where diarrhea is more severe, and results in higher morbidities and mortalities. We hypothesize that lactose-free milk intake during acute gastroenteritis would significantly decrease the duration and severity of diarrhea in infants presenting to the Emergency Department (ED), as compared with lactose-containing milk.

**Methods/Design:**

An open-label randomized clinical trial. Study population: 40 infants with acute gastroenteritis, age between 2 and 12 months, presenting to the ED, will be randomized to control or intervention group. Intervention: Lactose-free milk, whereas the control group will continue on regular infant formula for a total of 7 days. Infants will be followed up for 7 days. Outcome measures: Diarrhea duration, weight loss, illness clinic visits, hospitalization rate, parental satisfaction, and time to symptom resolution. Statistical analysis: Descriptive and regression analysis will be conducted under the intention-to-treat basis by using SPSS version 21.

**Discussion:**

Acute gastroenteritis is a public health burden for developing countries, with a significant impact on infant morbidity and mortality. Provision of infant formula that may reduce the duration and severity of diarrhea can decrease this burden in countries with limited healthcare resources, like Lebanon. The findings from this study are anticipated to provide evidence-based dietary recommendations for ambulatory infants with acute diarrhea in developing countries.

**Trial registration:**

ClinicalTrials.gov NCT02246010; September 2014.

**Electronic supplementary material:**

The online version of this article (doi:10.1186/s13063-015-0565-9) contains supplementary material, which is available to authorized users.

## Background

Acute diarrheal illness is a major public health burden worldwide, and more so in developing countries. Diarrhea is the second cause of morbidity and mortality in children younger than 5 years, accounting for around 15% of all childhood death, and leading to more than 1 million deaths each year [1]. The North American Society for Pediatric Gastroenterology, Hepatology, and Nutrition estimates the overall yearly incidence of diarrhea in children younger than 3 years at 1.3 episodes per child [2]. The mean incidence rate in developing countries is almost double that reported from industrialized countries and amounts to an average of three episodes per child per year [3]. Diarrheal diseases are also a financial burden at the macro- (country) and micro- (family) levels. In the United States, diarrhea leads to 2.1 to 3.7 physician visits per child each year, with an average cost of $289 per episode [4]. Moreover, pediatric diarrhea is known to afflict parental well-being, causing major emotional distress and fatigue.

Pediatric diarrhea can last for several days, leading to dehydration, electrolyte disturbances, and malnutrition, especially in children younger than5 years. Children who are malnourished, or have impaired immunity are at higher risk of life-threatening complications from diarrhea. The main etiology of acute diarrheal disease in children younger than 5 years is intestinal tract infection caused by a variety of viral, bacterial, or parasitic organisms. Acute infectious diarrhea may cause damage to the lactase-containing epithelial cells present on the tips of the intestinal villi, thus causing lactose malabsorption. The new epithelial cells that replace older ones are usually immature and often lack sufficient lactase activity, which will further exacerbate the lactose malabsorption, with subsequent prolongation of the diarrheal episode.

Adequate nutrition and hydration during diarrheal episodes is necessary to avoid dehydration, malnutrition, and weight loss. In a systematic review published in 1994, Brown et al. [5] reported clinical improvement after the use of lactose-free milk products in young children with severe dehydration. A recent Cochrane review reported the results of 33 randomized or quasi-randomized trials that assessed the effects of reducing or avoiding lactose in young children (<5 years) with diarrhea on the duration and/or severity of the illness [6]. The review concluded that lactose-free products may reduce the duration of the diarrhea by an average of 18 hours (MD, −17.77; 95% CI, −25.32 to −10.21, low-quality evidence) as compared with lactose-containing formula. Also, lactose-free products may reduce treatment failure, such as continued diarrhea or vomiting, continuing weight loss or need for additional rehydration treatment by around a half (RR, 0.52; 95% CI, 0.39 to 0.68, moderate quality evidence). However, all trials were conducted in high- or middle-income countries, and most of them included inpatients only. Hence, a need exists for high-quality trials to be conducted in developing countries where the incidence, severity, and consequences of diarrhea are high, including mortality and malnutrition.

In this study, we aim to investigate the effect of lactose-free milk on the duration and severity of diarrhea in infants with acute gastroenteritis in the ambulatory setting (Emergency Department). We anticipate that the findings of this study will add to the evidence base of dietary recommendations for infants with acute diarrhea in developing countries who are treated in ambulatory care.

## Methods/Design

### Study design

A randomized controlled open-label parallel-arm clinical trial to investigate whether lactose-free milk will shorten the duration and lessen the severity of diarrhea in infants with acute gastroenteritis, when compared with lactose-containing milk (Additional file 1).

### Study population

Infants who are fed artificial milk formula (2 to 12 months of age) with acute diarrhea, first seen in the emergency department (ED).

### Recruiting process

#### Inclusion and exclusion criteria

Eligible infants are those who are between 2 and 12 months of age, fed artificial milk formula, and who present to the ED of the American University of Beirut Medical Center with acute gastroenteritis. Diarrhea is defined as the passage of three or more loose or liquid stools in 24 hours, for at least 24 hours, and not exceeding 2 weeks from presentation, with or without fever, vomiting, mucus, or blood per stools.

Infants with any of the following conditions will be excluded: any breastfeeding, sepsis, chronic diarrhea, severe dehydration, inflammatory bowel disease, cow’s milk allergy, necrotizing enterocolitis, intussusception, volvulus, current intake of lactose-free milk, celiac disease, immune deficiency, chronic disease, malnutrition, or need of immediate hospitalization.

#### Randomization

Eligible infants will be randomly allocated to one of two parallel groups (experimental and control, 1:1 ratio), by using a computer-generated stratified block randomization. Stratification will be done by age groups: <6 months versus ≥6 months, with block sizes varying between 4 and 8. The random sequence will be generated by an independent biostatistician. Allocation concealment will be done to ensure that group assignments of the participants are revealed only after assessing that the inclusion/exclusion criteria are verified and consent obtained. This will avoid any bias that might be introduced by the investigator because of the knowledge of the allocation of the next subject. A set of sequentially numbered opaque sealed envelopes will be prepared with the allocation group, as per the randomization list, specified inside.

### Description of the intervention

#### Control group

Except for dietary instructions, subjects in the control group will receive standard medical care, including rehydration with oral rehydrating solutions, as per their physicians’ recommendations. The dietary instructions will be provided to the family by the research team and will consist of the infant’s regular lactose-containing milk, in addition to the hospital’s antidiarrhea diet usually prescribed to patients with acute diarrhea, and which is composed of potatoes, rice, lean meat or chicken, apples, bananas, and yogurt. Any additional treatment by the primary physician including dietary change (whether milk or solid foods) will be respected. If an infant in the control group does not improve on the lactose-containing milk, it will be up to his primary physician to decide on the next step, including switching to lactose-free milk. The infant will continue to be in the study and will be included in the intent-to-treat analysis. All dietary changes or medication intake during the study period will be recorded as part of data collection.

#### Intervention group

Infants in the experimental group will receive standard medical care, including rehydration with oral rehydrating solutions, as per their physicians’ recommendations. The dietary instructions will be provided to the family by the research team and will consist of the same antidiarrhea diet prescribed to the control group, as previously described, in addition to lactose-free milk (Similac LF). Any additional treatment by the primary physician, including dietary change (whether milk or solid foods), will be respected. If a baby in the intervention group does not improve on the lactose-free milk, it will be up to his primary physician to decide on the next step, including switching to any other lactose-free or lactose-containing milk.

### Outcome measures

#### Main outcome measure

The primary outcome is the difference in diarrhea duration between the intervention and control groups, defined as the number of days with loose or liquid stools, from randomization until the day of the last diarrheic stool passed.

#### Secondary outcome measures

Differences between the two groups with respect to the following:

1. Percentage weight loss from baseline.
2. Proportion of infants with a return physician visit because of diarrhea within 7 days from baseline.
3. Proportion of infants hospitalized within 7 days from baseline for any cause.
4. Parental satisfaction.
5. Severity of symptoms: stool quantity and quality, colic, lethargy, use of analgesic or antispasmodic.

### Recruitment

A trained research assistant will be present in the ED all day during regular working hours to identify eligible subjects. Parents of eligible infants will be approached and informed about the study, and details and procedure of the study will be explained to the family. If inclusion criteria are met, a written informed consent will be obtained from parents willing to enroll their infant in the study.

### Data collection

**Figure 1:**
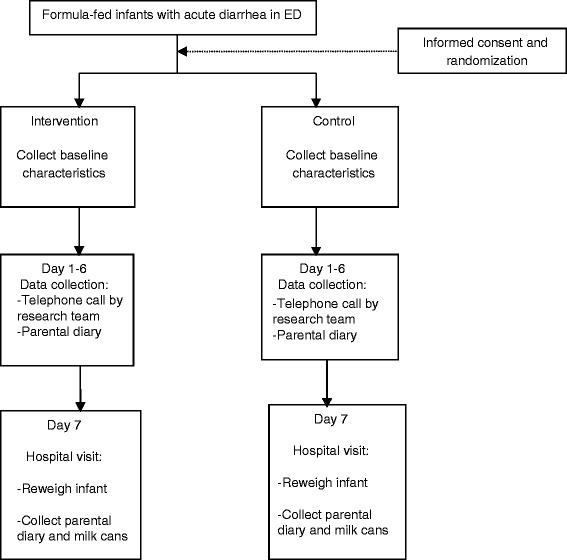
**A flow diagram of study participants.**

1. *Baseline:*
  - Obtain parental consent
  - Provide parent with dietary instructions, both verbal and written
  - Provide parent with milk formula as per treatment allocation
  - Provide parent with diary for data collection at home (see Additional file 2)
  - Collect baseline data: Age, gender, number of siblings, exposure to sick contact, day-care attendance, maternal level of education, maternal employment, maternal age, monthly income, smoking status at home, infant’s diet (formula brand, other dairy intake, oral rehydrating solution, antibiotic intake, vitamin/mineral intake, immunization status against Rota////virus, laboratory test results (if requested by ED physician, including blood, urine, or stool), number of stools per day, number of days with diarrhea, vomiting (number, duration), days with fever, analgesic intake, antispasmodic intake, history of previous breastfeeding, duration of breastfeeding, use of probiotics, initial diagnosis, physical examination findings.
2. Day 1 (after 24 hours): Telephone call to collect information on admission to hospital (Yes/No), return visit to doctor (Yes/No), and compliance with dietary instructions; reminder to fill diary.
3. *Day 3:* Telephone call to collect information on admission to hospital (Yes/No), return visit to doctor (Yes/No), and compliance with dietary instructions; reminder to fill diary.
4. *Days 5 and 6:* Reminders to fill diary.
5. *Day 7:* Visit to research team at AUBMC to collect information on admission to hospital (Yes/No), return visit to doctor (Yes/No), parental satisfaction, drug intake, final diagnosis; obtain infant’s weight on the same scale previously used at baseline; collect diary from parent, collect empty formula cans to check on compliance; pay transportation fees (See Figure 1).

### Data management and quality assurance

The research assistant (RA) will be trained on how to approach parents, contact them for follow-up by phone (by using a pre-paid phone card), and collect data during the 1-week follow-up. Training will entail familiarizing him or her with all necessary documentation, including enrollment and consent forms, and consenting in accordance with the ethical principles of informed consent. Instructions and demonstrations on how to explain the dietary instructions and fill the diary card will also be done. The RA will send daily reminders to parents to fill the diary by using email, telephone messages, or communication through social media.

### Sample size

We hypothesize that the lactose-free milk will shorten the diarrhea duration by an average of 24 ± 16 hours [6], as compared with lactose-containing milk. To detect this difference between the two groups with 90% power and 5% type I error, 12 infants will be needed in each arm (24 infants in total). We anticipate that 30% to 40% of infants that will be recruited will either withdraw or will be lost to follow-up from previous research experience. Hence, to allow for a loss-to-follow up rate of 35%, we will inflate the sample size to become 40 infants.

### Statistical methods

We will compare continuous variables (duration of diarrhea, percentage weight loss, age) by using the Student *t* test, and categoric variables (proportions, categories of severity) by using a χ^2^ test. Nonparametric tests will be used to analyze variables with skewed distribution or when the number of analyzed subjects is fewer than 30. Baseline characteristics of both groups will be compared to assure similarity of groups at study entry, and to identify clinically significant imbalance of any variable that needs adjustment during analysis. Risk ratios will be calculated for the outcomes of hospital admission and return physician visit. Results will be reported as means or medians when appropriate, intervals will be calculated. To assess the severity of diarrhea, we will evaluate the stool quantity and quality from the parental responses to the questions in the diary. For the quantity evaluation, we will consider passage of three to four stools per day as consistent with mild diarrhea, five to seven stools per day as consistent with moderate diarrhea, and eight or more stools per day as consistent with severe diarrhea.

For the quality evaluation, the responses will be assigned points ranging from a minimum of zero to a maximum of 6. A cumulative score will be created for the stool-quality assessment, which is the sum of points to all responses, ranging from zero to 14. We will consider that an overall qualitative score of 0 to 4 is consistent with mild diarrhea, 5 to 8 as consistent with moderate diarrhea, and a score of ≥9 as consistent with severe diarrhea. The main analysis will be based on the intention-to-treat principle. In addition, we will conduct per-protocol analyses for primary and secondary outcomes. The Statistical Package for Social Sciences (SPSS) version 21 will be used for data management and analyses. A *P* value of <0.05 will indicate statistical significance.

### Ethical approval

The study is approved by the Internal Review Board of the American University of Beirut. Written informed consent will be obtained from the parents. Because the study involves comparison of two types of milk formula, we estimate that the risks to infants from participating in this study are negligible, not exceeding those of current standard practice. Confidentiality of participants will be secured by storing data sheets in locked cabinets in the principal investigator’s office. Access to SPSS data file will be password-protected and restricted to the principal investigator.

## Discussion

This study aims at investigating whether lactose-free milk, when compared with lactose-containing milk, will shorten the duration and lessen the severity of diarrhea in infants with gastroenteritis who are treated in the ambulatory setting (ED) of a hospital in a developing country. The World Health Organization (WHO) estimates that 1.5 million children under the age of 5 years die each year of diarrhea; half of those are from the developing world [7]. Exclusive breastfeeding for the first 6 months of life is one cost-effective intervention that reduces the risk of infant death of diarrhea. Unfortunately, fewer than 40% of infants younger than6 months in developing countries are exclusively breastfed [8]. The lack of studies from the developing world investigating the effectiveness of lactose-free milk in reducing the duration or severity of infant diarrhea underscores the need for high-quality trials that can address this knowledge gap. Hence, this study may provide much-needed evidence regarding the dietary recommendations of formula-fed infants with acute gastroenteritis.

The study may have some limitations. The first limitation is the sampling frame, which is the ED of one hospital in an urban setting, from which a convenience sample of infants is recruited. It can be argued that infants living in rural areas may have a much more severe illness and may respond differently to the lactose-free milk. However, AUBMC is a major referral center for all the country, with patients seen in its ED from different urban and rural areas.

Another limitation is the small sample size. This is because the standard error used to calculate the sample size is relatively large (16 hours) in comparison with the estimated effect size (24 hours). Nevertheless, the effect size and its standard error were those reported by the most recent Cochrane Review about the subject.

The third limitation is the open-label design, which is due to the difficulty in concealing the milk containers in our setting, and to the fact that it is very likely that parents will decline participation in a trial in which they cannot know the name of the milk brand their infant will take. Despite these limitations, we anticipate that lactose-free milk, if proven effective, will have a significant impact on the morbidity of formula-fed infants with acute gastroenteritis in developing countries.

## Trial status

Recruiting as of November 1, 2014.

## Additional files

Additional file 1:
**SPIRIT Checklist. NA: Not applicable.**
Additional file 2:
**Parental diary for the diarrhea study.**

## Abbreviations

AUBMC: American University of Beirut Medical Center
ED: emergency department
SSPS: Statistical Package for Social Sciences
WHO: World Health Organization
