# Supplementary material for: Lactose-free milk for infants with acute gastroenteritis in a developing country: study protocol for a randomized controlled trial
Source: Trials. 2015 Feb 8;16:46. doi: 10.1186/s13063-015-0565-9 (PMC4324790; doi:10.1186/s13063-015-0565-9)
Supplement: Additional file 2: — Parental diary for the diarrhea study. [file 13063_2015_565_MOESM2_ESM.docx]

**Parental Diary for the Diarrhea Study**

**Day [__]**

***For questions 1, 2, 3 below, kindly describe the status of your child’s stools between 6 am yesterday and 6 am today***

1. The number of stools my child passed yesterday were [__[__]
2. Please describe the maximum amount of mucous observed in at least one stool yesterday:
3. None
4. Little (less than one fourth of stools)
5. Moderate (about half of stools)
6. Big (more than three quarters of stools)
7. All is composed of mucous
8. Please describe the maximum amount of blood observed in at least one stool yesterday:
   1. None
   2. Little (less than one fourth of stools)
   3. Moderate (about half of stools)
   4. Big (more than three quarters of stools)
   5. All is composed of blood
9. How many times did you give pain medicines to your child yesterday, if any?

(Please indicate names of all medicines)

Name of medicine:______________________________ [__[__] times

Name of medicine:______________________________ [__[__] times

Name of medicine:______________________________ [__[__] times

1. How many times did you give colic medicines for your child yesterday, if any?

(Please indicate names of all medicines)

Name of medicine:______________________________ [__[__] times

Name of medicine:______________________________ [__[__] times

Name of medicine:______________________________ [__[__] times

1. How many times did you give fever medicines for your child yesterday, if any? (Please indicate names of all medicines)

Name of medicine:______________________________ [__[__] times

Name of medicine:______________________________ [__[__] times

Name of medicine:______________________________ [__[__] times

***For questions 7 and 8, please indicate how much you agree with the below statements:***

1. My child’s activity was as usual most of the time yesterday
   1. Strongly disagree b. Disagree c. Agree d. Strongly agree
2. My child’s crying episodes were as usual most of the time yesterday
   1. Strongly disagree b. Disagree c. Agree d. Strongly agree

***For the next question, please use the stool chart below to describe your child’s stools.***

1. Yesterday, most of my child's stools looked like type [__]


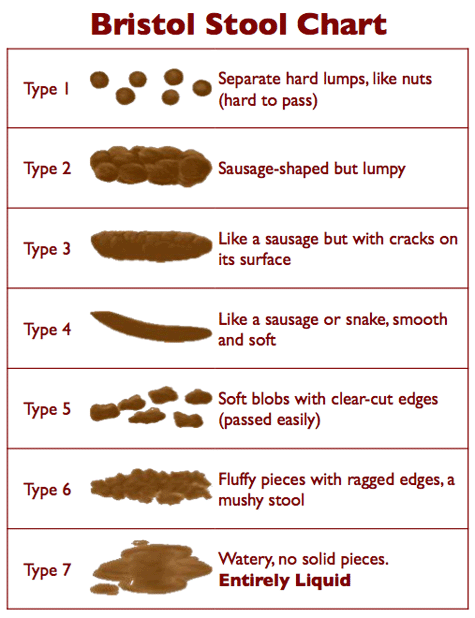


Adapted from Lewis & Heaton, 1997.

***For the next question, kindly record the amount of milk that your child drank between 6 am yesterday and 6 am today:***

- First bottle: ______________ ml.

- Second bottle: ____________ ml.

- Third bottle: ______________ ml.

- Fourth bottle: _____________ ml.

- Fifth bottle: _______________ ml.

- Sixth bottle: _______________ ml.

- Seventh bottle: ____________ ml.

- Eighth bottle: ______________ ml.
